# Understanding the educational needs of parenting athletes involved in sport and education: The parents’ view

**DOI:** 10.1371/journal.pone.0243354

**Published:** 2021-01-20

**Authors:** Masar Gjaka, Antonio Tessitore, Laurence Blondel, Enrico Bozzano, Fabrice Burlot, Nadine Debois, Dominique Delon, Antonio Figueiredo, Joerg Foerster, Carlos Gonçalves, Flavia Guidotti, Caterina Pesce, Andrej Pišl, Eoin Rheinisch, Ana Rolo, Gary Ryan, Anne Templet, Kinga Varga, Giles Warrington, Laura Capranica, Ciaran MacDonncha, Mojca Doupona

**Affiliations:** 1 Department of Movement, Human and Health Sciences, University of Rome Foro Italico, Rome, Italy; 2 Institut National du Sport, de l’Expertise et de la Performance, Paris, France; 3 CONI—Italian National Olympic Committee, Rome, Italy; 4 Faculty of Sport Science and Physical Education, University of Coimbra, Coimbra, Portugal; 5 University of Hamburg, Hamburg, Germany; 6 European Athlete as Student, Malta; 7 EUSA Institute, Ljubljana, Slovenia; 8 Sport Ireland Institute, Dublin, Ireland; 9 Ginàsio Clube Figueirense, Figueira da Foz, Portugal; 10 Department of Physical Education and Sport Sciences, University of Limerick, Limerick, Ireland; 11 University of Ljubljana, Ljubljana, Slovenia; 12 Sport and Human Performance Research Centre, Health Research Institute, University of Limerick, Limerick, Ireland; University of Birmingham, UNITED KINGDOM

## Abstract

**Background:**

Despite the fact that an educational programme for parents of youth tennis players has been launched recently, there is a need to empower parents of athletes in sustaining the combination of education and sport careers (i.e., dual career) of their talented and elite athletes across sports. The aim of this study was to explore the parents’ view of their role as dual career supporters and their need for educational support in this area.

**Methods:**

In total, 115 parents (F = 49, M = 66) of athletes (14–23 yrs) engaged in full time academic education (high school/university) and competitive (e.g., National, International) sports (individual = 12, team = 9) in five European Member States (e.g., France, Ireland, Italy, Portugal, Slovenia) took part in national workshops. The workshops involved discussing five themes and agreeing statements relevant to assist parents supporting their children as dual career athletes: 1) the athletes’ needs; 2) the sports environment; 3) the academic environment; 4) dual career-related policies and services; 5) The educational methods for parenting dual career athletes.

**Results:**

A final list of 80 agreed statements were identified: 25 statements mainly related to the sports entourage; 23 to information on dual career-related policies and services; 22 to the athletes’ needs; 17 to the academic entourage, and 8 to the relevant educational resources to parenting dual career athletes, respectively.

**Conclusions:**

This cross-national qualitative research synthesized the parents’ perspectives about their needs and the most relevant content of an educational programme for parenting dual career athletes. The findings of this research will help influence the formulation of effective education strategies on parenting dual career athletes to ensure an optimal supportive environment for the successful combination of high-level sport and education careers.

## Introduction

In Europe, competitive sports are mainly structured at a club level, with limited relationship with the educational system resulting in possible cultural and organizational divergences and potentially placing talented and elite athletes at risk of academic or sports dropouts [1]. In the last decade, the combination of academic and sport careers, referred to as ‘dual career’, of athletes has been considered as one of the priorities in European sports strategy and policy [2–5]. Such approaches have included funding provision for European Collaborative Partnerships between Member States [6, 7], a call for tender on minimum quality services for dual career [1, 8] and research for the Cult Committee of the European Parliament on qualifications/dual careers in sports [9].

Among the key stakeholders involved in establishing a supportive entourage for dual career [9, 10] and the opinion of athletes [11], parents have a tremendous and continuous role in encouraging and helping the athletes to commit, focus, persist, and efficiently organize their study and training [12–14]. The current literature has investigated the parental role in supporting their children coping with the demands of competitive sport participation, which encompass horizontal (e.g., transitions from youth to elite sports) and vertical (e.g., values, expectations, attitudes, etc.) critical stages [15, 16]. Empirical research highlights that parental views, goals, aspirations, styles, involvement, and monitoring of the educational pathway of the children are robust predictors of their academic achievements [17]. Nevertheless, few studies have collected information from parents themselves on their experience and perspectives as dual career supporters of athletes as students [18–26].

Parenting dual career athletes is a long-term process, often requiring more than a decade of involvement, spanning from the identification of a talented athlete to her/his developmental years and eventual elite sport stage as well as academic requirements at high school and university levels. Thus, dual career stages and transitions are characterized by significant variations in typology, volume, intensity, organization and planning of training and study demands, all which have an impact on the psychological, social and financial development of the athlete [27, 28]. In particular, parental support for student-athletes might be particularly relevant in countries with limited or no established policies and services aimed to help athletes combining their sports and education pathways [1, 9, 29, 30]. Therefore, parents’ attitudes, involvement and support in dual career could contribute to avoiding drop out when athletes are faced with the challenges of trying to balance a sporting and an academic career [31–34].

The extant literature providing information collected from parents themselves on their experience as key influencers and dual career supporters of athletes involve a limited number of participants from few countries and sports [12, 18–22, 24–26, 35–38]. In general, findings highlighted that the parental role provides the athlete with emotional, logistic and financial support, with parents perceiving themselves as not always prepared enough to understand, interpret, and play their role when interacting with the athlete and her/his sport and academic entourages. In light of the complexity of parenting dual career athletes, the need for specific education to enable positive parenting roles and fruitful relationships with athletes and other key stakeholders of the athlete’s sport and educational entourages has emerged [39–41]. Based on the scientific evidence on parenting of beginner tennis players [42–45], the International Tennis Federation recently launched an online educational programme for parents. Despite this initiative, there remains a dearth of information on the needs of parenting student-athletes from other sports. This emphasises the necessity to extend the knowledge and understanding of this phenomenon in relation to several sports and European countries [46].

To gain a thorough understanding of the educational needs of parents supporting the dual career of their talented and elite progeny, a consortium of ten European university and sporting institutions from six Member States (i.e., France, Ireland, Italy, Malta, Portugal, and Slovenia) engaged in a Collaborative Partnership to structure an Education Model for Parents of Athletes in Academics (EMPATIA), under the Erasmus+ Programme of the European Commission for the 2018–2020 period (590437-EPP-1-2017-1-SI-SPO-SCP). In including European countries spanning from dual career state-centric regulations (e.g., France and Portugal) to countries with laissez faire/no formal dual career structures (e.g., Ireland, Italy, Malta, and Slovenia) [29], the EMPATIA project intended to contribute to a wide understanding of the parents’ behaviours and the challenges of the parental role for an optimal support of dual career of athletes [47]. The EMPATIA project was designed to increase the knowledge, understanding and awareness within the scientific community of the unique challenges facing parents of dual career athletes and to provide a scientific evidence base for the development of appropriate educational resources to support parenting dual career athletes. EMPATIA utilises concept mapping methodologies to gather, synthesize and evaluate the needs and challenges experience by the parents of talented and elite dual career athletes. Concept mapping is a systematic methodology based on a predefined sequence of research phases designed to organize and represent ideas through its unique integration of qualitative and quantitative methods [48, 49]. In particular, in order to construct a concept map, a wide variety of relevant stakeholders have to generate statements through group discussion [48]. Indeed, qualitative methods could be useful when seeking to systematically describe and explain the parents’ views [43] regarding dual career. In having participants discuss and uncover specific topics in common with each other, focus groups could provide a valuable method to pursue in-depth exploration of this topic and to provide insights into how parents of dual career athletes perceive their role [50]. In fact, focus groups assign a central role to participants and a peripheral role to the researcher/observer, who have to ‘facilitate/moderate’ group interactions and in-depth discussions via a logical sequence of open-ended questions on specific topics, to encourage a universal participation within the group, and to pay particular attention to the ‘sensitive moments’ of group interaction (e.g., more or less dominant speakers and silences) ensuring a relatively balanced contributions of participants [50].

Therefore, the purpose of the study was to investigate through the use of focus groups the current experiences of student-athletes’ parents and their educational needs to support their parental role. It was hypothesized that parents might face various difficulties (e.g., financial, emotional, logistical) in supporting the student-athletes, in connecting with different dual career constituents (e.g., other children, parents, sports staff, and academic staff), and in being informed on the dual career policies and best practices in place in the countries where the student-athletes live or in other countries where they intend to relocate for sport or academic purposes.

## Methods

### Design and methodology

The purpose of the focus groups that we conducted with the parents of dual-career athletes was to enable participants to collectively stimulate, build upon, and question ideas and concepts through discussion [51], with the desired outcome of generating a consensus on a series of statements describing their issues, problems, concerns and needs. This consensus was then used as the basis for subsequent concept mapping [48]. The assumptions that framed this research mainly follow an eminence-based approach, but also an evidence-based approach grounded on an initial systematic literature review that provided insight into five main themes.

When using qualitative research methods [52–54] in a preliminary phase of analysis, the goal is to identify the definitions of reality that inform the experiences of research participants. In particular, the thematic analysis of a systematic literature review provided insight into the challenges faced by parents as they provided support for their dual career athletes [55]. Our interpretive analysis data identified five major themes around which the parents experienced challenges. They were their: (1) relationship with the athlete; (2) relationship with the sport environment; (3) relationship with the academic environment; (4) approach to both sport and education; and (5) stressors and coping. These themes were then used as a basis for conceptualizing relevant research questions and doing an interpretive analysis of qualitative data [55]. Accordingly, the research team created five specific questions to identify the types of education needed by parents of student-athletes: 1. What aspects the athletes need to be supported in their dual career?; 2. What information relative to the sports environment is necessary for helping parents support the athlete’s dual career?; 3. What information on the academic environment is necessary for helping parents support the athlete’s dual career?; 4. What information on dual career-related policies and services are necessary to assist parents as they support the athlete’s dual career?; and 5. What educational resources are relevant to educate parents as they support the athlete’s dual career?

Finally, to understand the role of parents in supporting student-athletes in their dual career paths over time, Bronfenbrenner’s four systems of influence (i.e., *person*: mother, father, or guardian role; *process*: how parents/guardians interact with the sport and academic environments of their child; *context*: dual career policies in place at the meso and macro dual career dimensions; and *time*: academic and sport levels of the student-athletes) were used as a theoretical framework for interpretation and understanding [56].

To enhance the rigor with which our research was executed, the research team developed guidelines for National focus groups with parents of talented and elite dual career athletes, which outlined the defined research questions, the purposeful recruitment of participants, the focus group standard operating procedures, the data collection and synthesis, and the instructions to be provided to the parents participating in the focus groups. Thus, eight criteria for qualitative excellence were met [52, 53]: 1) worthy topic, based on dual career research [9–11, 55]; 2) rich rigor, grounded on developed guidelines for National focus groups with parents of talented and elite dual career athletes, in which the research questions, the purposeful recruitment of participants, the focus group standard operating procedures, the data collection and synthesis, and the instructions to be provided to the parents participating in the focus groups were clearly defined; 3) sincerity, built on open discussion between the members of the research team in designing questions, in conducting focus groups without interfering with the participants’ opinions, and in analysing data without a judgemental attitude; 4) credibility, to foster multiple opinions and substantiated by the various perspectives represented in the outcomes of the focus groups; 5) resonance, based on the involvement of parents of talented and elite dual career athletes, which per-se amplifies the actual outcomes of dual career support; 6) significant contribution, based on the knowledge that current findings represent the baseline for the following concept mapping procedures, as well as precious insights for parents of student-athletes, scholars, and policy makers; 7) ethical, based on the Declaration of Helsinki criteria, and certified by the external approval of the European committee selecting the EMPATIA project and the approval by the Research Ethics Committee of the University of Ljubljana of the EMPATIA project (9:2018), which included the organization of national focus groups in five countries (i.e., France, Ireland, Italy, Portugal, and Slovenia); and 8) meaningful coherence, achieved through the opinion of European dual career experts on the coherence between the research aims, procedures, outcomes, and interpretation.

### Participants

To ensure an appropriate representation of parents aligning with agreed inclusion criteria define by the EMPATIA project team a purposeful sampling was deemed appropriate and a core strength to gain comprehensive meaningful and practical knowledge on parenting dual career athletes [50]. Thus, potential participants were identified by screening the National sports organizations’ databases, using the following inclusion criteria: athletes’ age (range: 14–24 yrs), sex (e.g., female or male), type of sports (e.g., individual or team), competitive level (e.g., national and international), and academic level (e.g., high school or university). Subsequently, an invitation and consent letter was sent to male and female parents who were presumed to provide the best data to address the purposes of the research.

In total, 115 parents (49 mothers, 66 fathers; France: n = 20; Ireland: n = 17; Italy: n = 26; Portugal: n = 16, and Slovenia: n = 36) of athletes competing in 12 individual (e.g., athletics, canoeing, dancing, diving, equestrian, fencing, gymnastics, kayaking, modern pentathlon, sailing, shooting, swimming) and 9 team (e.g., basketball, handball, hockey, rowing, rugby, synchronized swimming, soccer, volleyball, water polo) sports, provided written consent to obtain information on how they perceive their parental role and challenges, and their perceived needs in terms of an educational support for parenting dual career athletes. To maintain a manageable sample size for in-depth qualitative discussion [57], 12 focus groups were organized, each lasting 2 hours.

### Procedures

At the beginning of the focus groups, the organizers provided a 5–10 min presentation on the EMPATIA project and its aims, the expected contribution of participants, and the standard operating procedures of the focus group. Then participants were organized around a table in sub-groups of 5 people with the question to be addressed clearly written on a flipchart. One parent was assigned a leading position, with a researcher as facilitator acting and an observer manually registering supplementary observational data (e.g., context, environment, personal gesture, posture and the like). The open-ended questions allowed parents freedom to interact directly, questioning one another, recalling personal anecdotes, building upon one another’s views, sharing personal experiences, and agreeing or disagreeing with opinions. Each participant was encouraged to write on the flipchart the statements regarding the most important factors considered relevant for answering the question. After a 15-minute discussion, the sub-group leaders had to report the statements to all participants and a 5/7-minute general discussion was allowed. Then, the composition of the sub-groups was changed. When all the five questions had been addressed, a 20/30-minute general discussion with all the participants identified and agreed a final list of statements from their focus group deemed relevant to parenting dual career athletes.

### Data analysis

Any statement (e.g., word, short phrase or sentence) emerging from each of the five research questions (i.e., the content units) during the National focus groups was recorded. To produce a conceptually and semantic equivalent translation of the original statement, two members of the research team from each country performed independent translations in English and agreed on a combined version, which was subsequently backward translated by an English reviewer using a blind translation procedure. According to the literature [58], the best approach to the analysis of focus group data should be consistent with the original purpose of the research and the information needs. In considering that complete content unitization becomes very difficult when multiple focus groups are carried out on the same general topic in different countries, content units (e.g., the syntax or structure in which a registered unit/statement occurred) were deemed a basis for interpreting and organising the recorded statements. At a consensus meeting of the EMPATIA project team (N = 20) repetitions within and between each of the five content units were eliminated; fragmented statements were condensed into related broader statements; the clarity and comprehension of all factors was improved and the relevancy of each factor to the aim of the EMPATIA project was confirmed.

To validate the clarity of the statements, during the 2018 European as Athlete conference on dual career, 32 dual career experts from 11 countries (i.e., Belgium, Brazil, Bulgaria, Croatia, Hungary, Italy, Latvia, The Netherlands, Portugal, Spain, and Sweden) were requested to score the list of statements by means of a 5-point Likert scale (ranging from 1-not clear at all, to 5-very clear), with a clarity threshold ≥ 4.0 pt. In light of the potential cultural differences within the European contexts, the consensus method was deemed especially important to maintain the collegiality of decisions [59].

## Results

From the 211 descriptive statements of parenting dual career athletes established in an open-ended and non-judgmental fashion, a list of 80 statements was synthesized, validated, and analysed. Table 1 presents the 80 statements organized in the five content units and related sub-themes.

**Table 1:** List of statements to guide the development of an Education Model for Parents of dual career Athletes in relation to the five content units.

EMPATIA Statements to guide development of Education Model for Parents of Dual Career Athletes	Athlete's needs	Sports entourage	Academic entourage	Policies and services	Educational resources
14.P/G understanding of their co-parenting role and support of the athlete.	parental values				
43.P/G awareness and understanding of sport as a way of life	parental values	parental role			
54.Father/male guardian perspective of the importance of the athlete’s success in education & sport	parental values				
60.Mother/female guardian perspective of importance of the athlete’s success in education & sport	parental values				
65.Athlete valuing their DC status as a way of life	parental values				
67.Clarify P/G role in supporting athlete in long-distance travel and intercultural experiences	parental values				
68.Knowledge for the P/G to make a long term plan for the sport and education career of the athlete	parental values				
6. Information for P/Gs on athlete nutritional needs and available supports	health/wellbeing				
8. Ongoing evaluation of athlete life style	health/wellbeing				
11.Ongoing evaluation of athlete mental health and wellbeing	health/wellbeing				
39.Information for P/Gs on athlete psychological needs and available supports	health/wellbeing				
48.More information for P/G regarding rest, injury management and rehabilitation of the athlete	health/wellbeing				
51.P/G understanding/resolving athlete’s risky behaviours e.g. crises, alcohol & drug use, gambling	health/wellbeing				
63.Information for P/Gs on athlete medical needs and available supports	health/wellbeing				
70.Understanding impact of DC on socialisation & social interactions of athlete e.g. siblings/peers	health/wellbeing				
75.Ability of P/Gs to monitor the signs and symptoms of athlete wellbeing	health/wellbeing				
78.Preparing P/Gs to deal with emotional response of athlete to sport performance	health/wellbeing				
1.Ability of athlete to effectively manage DC time e.g. school, study, sport	logistics/time mamagement				
37.Information for P/G on financial management skills relevant to the student-athlete.	logistics/time mamagement				
46.Access to/appointment of national DC coordinator to support the needs of athletes	logistics/time mamagement				
49.P/G skills to manage the family time commitment/logistics for athlete e.g. transport/time/roles	logistics/time mamagement				
74.Ability of athlete to effectively manage independent living e.g. cooking, cleaning, resting	logistics/time mamagement				
12.Knowledge of how and when P/Gs can join the athlete during training/competition.		parental role			
23.Clarify the role of the P/G in the sport environment		parental role			
30.Setting of realistic & achievable goals for P/Gs & athletes in sport and educational environments		parental role	school/university		
35.Communication skills/pathways for P/G to assist the athlete sport career e.g. speaking to coach		parental role			
56.Representation of P/Gs at sport administration/federation levels		parental role			
73.Clarify role of father/male guardian for athlete in absence of supports/quality coaching		parental role			
77.Clarify role of mother/female guardian for athlete in absence of supports/quality coaching		parental role			
22.Mentoring for athlete in both sports and education environments		club/federation	school/university		
26.Develop list of DC friendly schools and universities		club/federation		actual policies/provisions	
27.Utilising sport clubs as hubs for DC information sharing and updates		club/federation		provisions	
3.DC support providers in **each** sport federation		club/federation			
31.Provision of sport calendar to facilitate planning for educational career		club/federation			
32.Information for P/Gs on selection procedures within sports		club/federation			
33.Sport partnerships with industry/business to support DC		club/federation		cooperation and partnerships	
36.Information for P/Gs on international DC policies/services available in the sport environment		club/federation		actual policies/provisions	
40.Information for P/G on national procedure/service/opportunities for athlete in sport environment		club/federation		actual policies/provisions	
45.More communication to P/G from sport environment e.g. athlete progress/ranking/injury/training		club/federation			
55.Information for P/Gs on financial cost/support in sport environment e.g. equipment/travel costs		club/federation			
57.Access to quality coaching for athlete along the DC pathway		club/federation			
66.DC awareness and support from the coach		club/federation			
7. P/G basic knowledge of the various aspects/demands of the sport		club/federation			
71.Information for P/Gs on how co-operation between sport and educational environments takes place		club/federation	school/university		
80.Access to DC help desk/contact person available in sport environment		club/federation		provisions	
9.Support/guidelines for P/G & athlete on conflict prevention /solution with **sport environment**		club/federation			
10.Communication skills/pathways for P/Gs to assist athlete education career e.g. speaking to tutor			parental role		traditional means
15. Increased awareness of the needs & circumstances of the athlete in the educational environment			parental role		
64.Clarify role of P/G in the education environment e.g. homework support/negotiate for athlete			parental role		
29.Support/guidelines for P/G & athlete on conflict prevention/solution with education environment			school/university	new policies	
38.Distance education provision for athletes e.g. at training centres /when travelling due to sport			school/university	provisions	
41.P/G information on financial cost/support in education environment e.g. fees/housing/scholarships			school/university	actual policies/provisions	
72.Access to DC help desk/contact person available in education environment			school/university	provisions	
5. Information on the quality of schools from educational perspective			school/university		
24.Advice for athlete on high school and university course selection.			school/university		
28.Information for P/Gs on relevance of education/degree for future opportunities of athletes			school/university		
50.P/Gs feedback regarding educational progression at university (agreement of athlete required)			school/university		
58.P/G information on national procedure/ service/opportunity for athlete in education environment			school/university		
61.Information for P/G on educational supports for the athlete e.g. class notes/flexible deadlines			school/university		
62.Provision of education calendar to facilitate planning for sport career			school/university		
4.Co-operation between sport (e.g. club and federation) and education sectors in DC needs				cooperation and partnerships	
13.Sport partnerships with educational institutions to support DC				cooperation and partnerships	
17.Knowledge and exemplars of national and international DC best practice.				actual policies/provisions	
19.Information on potential to adapt educational schedule to accommodate demands of sports career				actual policies/provisions	
20.Information on potential to adapt sport schedule to accommodate demands of educational career				actual policies/provisions	
44.Provide regular updates to P/Gs on changes in policy, services and support for athletes				actual policies/provisions	
53.Information for P/Gs on international DC policies/services available in education environment				actual policies/provisions	
16.Recognition of prior learning outcomes and sport experience for educational credits.				new policies	
21.Policy to support athletes at end of sport career				new policies	
52.Inclusion of non-elite/talented athletes in DC athlete legislation				new policies	
59.Development of national policy to formally recognise student-athlete (Dual Career) status				new policies	
76.Good practice guidelines for P/Gs to support the athletes				new policies	
79.Development of international policy to formally recognise student-athlete (Dual Career) status				new policies	
2.Technology/social media knowledge for P/Gs to access educational information relevant to athlete					technology/social media
25.Technology/social media knowledge for P/Gs to access sport information relevant to athlete					technology/social media
34.E-Learning Opportunities: P/G access to DC social media networks, interactive forums & websites					technology/social media
18.DC educational brochure for P/Gs which includes polices, opportunities, services etc					traditional means
42.Best practice presentations by DC athletes and P/Gs of athletes					traditional means
47.Identification of successful student-athletes who can act as ambassadors for dual career					traditional means
69.DC workshops for P/Gs on education, sport & policy environments e.g. supports/best practice/law					traditional means

Relative to the five content units, the research team assigned 25 statements to the sports entourage, 23 to information on dual career-related policies and services, 22 to the athletes’ needs, 17 to the academic entourage, and 8 to the relevant educational resources to parenting student-athletes, respectively. Fifteen statements (i.e., number 10, 22, 25, 25, 29, 30, 33, 37, 38, 40, 41, 43, 71, 72, and 80) loaded on two content units. For the athletes’ needs, 10 statements (i.e., number 6, 8, 11, 39, 48, 51, 63, 70, 73, and 78) pertained to aspects related to health and wellbeing, 7 statements (i.e., number 14, 43, 54, 60, 65, 67, and 68) related to values, and 5 statements (i.e., number 1, 37, 46, 49, and 74) concerned life management and logistics. Statements associated with the sports entourage referred mostly to the sport clubs and/or federations (i.e., number 3, 7, 9, 22, 26, 27, 31, 32, 33, 36, 40, 45, 55, 57, 66, 71, and 80), whereas 8 statements (i.e., number 12, 23, 30, 35, 43, 56, 73, and 77) related to the parental role. Out of the 17 statements linked to the academics entourage, the majority of them concerned the schools and universities (i.e., number 5, 22, 24, 28, 29, 38, 41, 50, 58, 61, and 62) and only 4 statements (i.e., number 10, 15, 30, and 64) referred to the parental role. For dual career-related policies and services included several statements requesting more information on existing policies/provisions (i.e., number 7, 19, 20, 21, 26, 36, 40, 41, 44, 53, and 59,) or envisaging new policies (i.e., number 16, 29, 52, 76, and 79), whereas 4 statements referred to requests of provisions (i.e., number 27, 38, 72, and 80) and 3 to needs of dual career cooperation/partnerships (i.e., number 4, 13, and 33). Finally, for the relevant educational resources for parenting dual career athletes, statements related to information technologies/social media were less represented (i.e., number 2, 25, and 34) with respect to more traditional educational means (i.e., number 10, 18, 42, 47, and 69).

## Discussion

The current study contributes to foster the dual career culture by presenting the views of 115 European parents of dual career athletes competing in 21 sports in five European Member States, which differ in terms of history, culture, economy, sport preferences, and dual career policies in place. In including countries with well-established structures and policies required to assist athletes and others with fragmented dual career policies [29], this research work advances the academic knowledge and provides broad insights into the parents’ views and perceptions regarding their educational request for optimizing their relevant role in support of dual career athletes competing in different sports. In fact, a sound understanding of the actual rather than assumed needs for parental support is essential to generate bespoke guidelines for the organization of customer-based dual career education programmes for parents [47, 60]. In the current work, the interaction between parents from several sport contexts indicated that the collective view of parent’s needs of education concerns several aspects of dual career, highlighting the complexity of the parental role in supporting student-athletes. The distribution of key aspects was comparable between the themes related to the athlete’s needs, the parental relationships with the sport and academic entourages, and information on dual career policies and provisions. Furthermore, the fifteen statements loading on more than a content unit, substantiate the complex dual career processes that parents experience in the development of their crucial supportive role of student-athletes [56].

Furthermore, both traditional and technological resources for dual parenting education were identified.

### Parental support of the athletes’ needs

In this study, parents recognized the need of an education on parenting student-athletes to attain a clear awareness of their parental role and perspective in guiding the athletes’ choices in education and sport careers. In fact, parents recognized that they play a crucial role in providing motivation, encouragement, and support when prioritizing relevant athlete-centred informed short- and long-term plans for sound educational and sport decisions, which could have immediate and/or longer-term implications. In fostering expectations of continuing education viewed as non-negotiable, parents contribute decisively to the formation of the athletes’ attitudes towards dual career that help them not questioning their dual paths, maintaining their motivation to achieve in both domains, being resilient in case academic obligations conflict with their sport duties emerge and extract their potential both in athletics and academics [21, 36, 38, 61].

A holistic perspective is needed to assist the student-athletes coping with positive and more challenging aspects of their individual dual career pathways [62–64]. To enhance their self-awareness and readiness to be optimal supporters of student-athletes, the participants in this study showed a particular interest in the need of receiving information on the health and wellbeing of their talented and elite athletic progeny, including actual health status and health-related needs, injury prevention and rehabilitation, lifestyle monitoring for the prevention and resolution of emotional responses, possible crisis to positive/negative athletic outcomes, and risky behaviours, as well as on possible performance-enhancing nutritional and psychological support [19, 24, 36, 40]. In light of several health-related (e.g., unhealthy dietary habits, use of illegal supplement, alcohol, drug, etc.) and psychosocial-related (e.g., bullying, depression, gambling, match-fixing, violence, sexual harassment, etc.) risky behaviours reported in athletes [65–68], the availability of performance lifestyle and/or welfare advices could help parents supporting their daughter/son through her/his sport experience [22].

Despite the literature claiming that parents have a consistent readiness to provide a solution-focused logistical support for the student-athletes [12, 22, 23], the present findings indicate a need of specific education on parental skills to manage their personal, family and athlete’s time, as well as financial aspects. In accordance with the existing literature [19, 36], parents reported that the time spent for travelling to training and competitions could affect their overall family life and their relationship with other children who might perceive receiving an unequal parental dedication, and might also conflict with their work commitments. Furthermore, they mentioned economical aspects due to academic and/or sport fees, and expenses related sport equipment, participation to competitions, transport, accommodation, equipment lessons expert coaching, academic support, and extra academic lessons. Without a formal economic support at academic and/or sport levels, talented athletes might be precluded from progressing in their dual career path [19, 23].

To help them supporting the needs of the athletes and their family, some parents envisaged an access to a national dual career coordinator with high levels of contextual intelligence within this domain. Indeed, a cooperation with a dual career specialist, who would serve as a point of contact, is strongly envisaged to help parents learning to recognize early symptom and effective strategies to resolve the problems the athlete is experiencing in the sport, educational and social contexts [22, 40, 69], and consulting on the balance between support and encouragement of independence [70]. Actually, the presence of nationally accredited specialists committed to promoting and enhancing the health of the student-athlete is considered a minimum requirement for dual career service providers [1]. In this respect, there is a need of academic institutions and sport organizations that recognize the complex challenges of dual career paths and cooperate to shape a friendly dual career environment [1].

### The parents and the sport entourage of the athlete

The sport entourage encompasses several actors (e.g., coaches, physical trainers, team, club and sport managers, etc.) who might perceive problematic the involvement of parents in sport-related decision-making, especially in presence of differences in cultural, educational, and social background [60]. Despite several scholars claimed that meaningful relationships between coaches and parents are essential prerequisites for aligning objectives to promote a holistic developmental process of talented athletes [22, 69, 71–73], in practice the sport entourage tends to attribute a limited consideration to the parents.

In this study a need of specific education on the parent’s roles, presence, and function at sport level emerged to set realistic and achievable dual career goals, especially when quality coaching is lacking. To foster the advancement of awareness of dual career related-issues, provision of dual career tutorship, mentoring, services, and opportunities, parents envisaged their involvement in the decision bodies of sport clubs and sport federations. In fact, the sport system tends to be resilient to take responsibility in dual career, considering educational aims secondary or problematic for high-performance [1, 74, 75]. Conversely, the parents expected the clubs/training sports centres to become potential hubs for information sharing on specific sport contexts, demands, calendars, talent development programmes and processes, as well as on the existence and availability of agreements with sport-friendly academic institutions and partnerships with the business sector. In fact, student-athletes supported by sport organizations with effective capability to negotiate with educational bodies admission procedures, examination schedules and tutoring tend to show high motivation towards their academic career [76]. Finally, a continuous communication regarding the athlete’s selection, progress, training and health status was deemed essential to generate sound educational and sport short- and long-term plans. Therefore, parents highlighted the need of a positive interaction with coaches for athletic and academic achievements, especially in considering that the coach becomes a more prominent social agent and role model as the athlete ages [21] and that coherence between coaches and parents would encourage athletes to endure education as well [22]. In fact, coaches have an instrumental role not exclusively related to the mastery of sport-specific skills but also in the overall holistic development of athletes and their decision-making at every stage of their careers [61, 72].

Several of the aspects emphasized by parents have been recommended to sports club/training sport centres to increase their awareness in dual career with respect to parents, education, business, and their own coaches [69]. Furthermore, the cooperation between parents and sport entourage is vital to build common perspective and prospective views of dual career to be shared with the athlete and other dual career stakeholders at academic and/or labour market [46]. In this context, the International Tennis Federation recently launched an educational programme for parents to enhance their support of youth tennis players and to improve their involvement in this sport setting.

### The parents and the academic entourage of the athlete

Similarly, to the sport entourage, the academic system should not care only for professional knowledge (e.g., subject matter and curricular knowledge), but also attempt to be adaptable for developing multi-culturally educated and engaged citizens in an era of globalization [77]. In general, the transition from secondary school to higher education might parallel that from youth to elite sports, both posing several challenges due to increased demands. In recognition that Member States rule differently in sport and education, in relation to dual career the European Commission has not proposed a one-model approach in support to higher education for athletes [3]. However, depending on the local cultural, contextual and organizational aspects, athletes might need different degrees of flexibility from the educational systems, which do not imply “watered down” academic courses. Arguments against policies on particular attention to extracurricular sport participation of students often concern the specific adjustments to the academic programs and the extra care and time of teachers to meet the unique requirements of student-athletes. The extent of the teachers’ cooperation is based on their individual dual career views and personal interests rather than on the institution’s dual career vision, strategy and policy [1, 23], with some university professors being positively inclined to make personal agreements with athlete to create flexible educational provisions even in absence of established policies [78].

Previous research emphasises a lack of consistency in educational support (e.g., individualized study plans, supplementary classes, electronic diary of daily school activities, exam arrangements, e-learning, tutorship, career orientation, scholarships, part- or full-time boarding, professional coaching, sport facilities, medical, paramedical and psychological support) between and within Member States [1, 79]. In the present study, parents highlighted a need of information on procedures, services and opportunities provided by athlete-friendly high schools and universities, which is important to guide knowledgeable educational decisions for future opportunities of athletes. Furthermore, the parents demanded a specific education to clarify their role in: a) negotiating for the athlete within the education environment; b) helping the athlete setting realistic goals at academic and sports level, and; c) increasing their awareness of educational needs and circumstances and prevention of conflict within the education environment. Finally, the presence of mentors and tutors was envisaged to assist a fruitful liaison between teachers, parents and coaches about any pertinent issues concerning the athletes in relation to the education and sport calendars. In fact, athletes appreciate tangible and informational support for a constant communication between clubs, school and parents [22]. In this respect, the outcomes of two recent European Collaborative Partnerships on dual career tutorship [80] and support providers [81] could be valuable resources.

### Information on dual career policies and services

European strategies and approaches to dual career are still in a developmental phase and require a continuous dialogue and exchange of best practices between stakeholders for further implementation, which should include the contribution of parents who are well placed to present clear insights into the support needs of the athletes. In general, the parents involved in the current study were unaware of the European guidelines, policies, services and best practices in place in other Member States. Interestingly, almost half of the statements included in this content unit loaded also on other content units, with a series of issues discussed previously in the sport (n = 6) and educational (n = 4) entourages. These findings emphasize the deep link between legislative regulations and practical actions in the parents’ view of dual career.

A compelling demand for cooperation between academic, sport, and labour market emerged from several statements, reiterating also the European recommendations in dual career [2–5, 8, 9, 30], and the advices for minimum requirements of dual career services [1] and sports clubs [69], which fostered new dual career agreements, policies and services in several Members States. However, not all the academic institutions are sensitive to adapt educational programmes (i.e., scheduling) to accommodate the demands of sports career, whereas sport bodies result even more resilient to accommodate to meet the academic demands. It could be hypothesized that a regulated cooperation between these systems could determine new and effective ways to overcome their actual rigidity. Through its annual conference, networking activities, research, and cooperation with European projects, the European Athlete as Student network (www.dualcareer.eu) represents the knowledge hub for educational bodies (i.e., universities, high schools, sports schools) and sport organisations (i.e., clubs, federations, Olympic committees) involved in envisaging, advancing, and putting into practice the advancements dual career culture [10].

To guarantee a full recognition of athletes as students at national and international levels, parents highlighted the need of a clear definition of dual career status. This aspect is very critical because different requirements and eligibility criteria for dual career programmes and services determine unequal treatments of elite European student-athletes, especially those who migrate for sport and/or academic purposes in a Member State different from their native one [1, 9]. Parents also demanded dual career legislation inclusive of talented-but not yet elite athletes. In fact, for achieving an elite status a long process of skill development is needed, during which talented athletes might be at risk of sport or academic dropout [82]. Finally, the recognition of learning credits deriving from sport experience has been demanded. In fact, sport participation is considered relevant for the acquisition of several ‘soft skills’ in the emotional, cognitive, social, and psychosocial domains that could be transferred across settings [83]. However, to be considered viable, this request needs a thorough understanding, recognition and formalization of the knowledge and skills acquired through sports.

### Educational resources for parenting dual career athletes

Educational resources encompass traditional face-to-face and self-directed web-based delivery education, the latter used to reach out large audiences, to eliminate logistical constraints, to allow learning by repeating activities and review educational content at an individual pace [84]. In light of these considerations, face-to-face education for parents of dual career athletes might be considered less appealing. However, in the present study the parents valued printed educational material, and life interpersonal interactions (e.g., presentations, seminars, and workshops with dual career experts and successful former student-athletes), which do not require technological competence and favour communication skills. Furthermore, online discussion forums and websites have been envisioned. These findings are in agreement with the literature on a web-based educational programme for parents of tennis players [60], which reported that parents and less interested in joining web conversations in forums and perceive more effective face-to-face interactions with other parents, probably being used to meet them during training sessions and competitions. As indicated by the participants in the current study, sport clubs and academic institutions should seriously consider the quest of parents to function as dual career hubs and start organizing discussion groups, parent-staff meetings, or peer mentoring programs. In fact, “blended learning” could be as an interesting opportunity for sport parenting education [60] and for establishing a strong alliance between dual career actors [69].

### Limitations and future directions

Whilst a quantitative approach to nomothetic theory generation usually results in highly generalizable theories, a qualitative approach is better suited to generate mid-range theories through a hermeneutic path that allows balancing generality and specificity of the generated assumption [85]. The present eminence-based approach based on qualitative methods led to the identification of theory areas appropriate to frame the focus group issues and to derive mid-range theories that are sufficiently generalizable, but also sufficiently specific, to inform the development of good athletes’ parenting strategies and practices. In collecting the parents’ views and perceptions, the present findings provide a thorough identification and categorization of relevant factors to be included in a tailored educational programme for parents of dual career athletes.

This broad knowledge in the understanding the educational needs of parents of student-athletes uncovered the complex phenomenon of parental support for athletes but two main limitations are still present. First, despite the adopted eminence-based approach did offer possible connections between factors, it is important to bridge the fundamental gap of considering relevant factors as standing on their own by means of systematic way of compiling and synthetizing this qualitative information is needed to manage and frame it in contextual and cultural lenses. In particular, further research should consider a concept mapping methodology involving a system-based approach to integrate ideas and knowledge across stakeholder, which could be valuable to improve theory development as a sound basis for directing not only effective educational programmes but also dual career policies and interventions [86, 87]. Thus, the identified statements will form the basis for a sequence of robust operationally-defined concept mapping steps to yield a conceptual representation of the education for parents of dual career athletes [48, 49]. In fact, to advance our knowledge on specific ways in which these factors relate, there is a need of meaningful research breakthroughs in the understanding of distinct clusters of factors related to parenting dual career athletes, which could improve theory development as a sound basis for educational programme development.

Second, the data from the present research are intended as a base to design an EMPATIA educational programme for parents of student-athletes. However, in considering that parenting dual career athletes is a complex multifaceted phenomenon, it is conceivable that the limited sample of dual career experts, university professors, coaches, and club directors from six European Member States would develop educational material not necessarily fitting the needs of parents of student-athletes across and beyond Europe. Thus, further research should consider the opinions of a representative cohort of parents piloting a specific programme for parenting dual career athletes and providing insights for future revisions.

## Conclusions

The current cross-national qualitative research synthesized the parents’ perspectives about the needs of an education for parenting dual career athletes, which are not available in studies carried out in a single country. The present findings highlight four key dual career partners: the student athlete, the academic staff, the sport staff, and the parents themselves, who are often inexperienced regarding operational strategies and have limited information on specific dual career policies and services currently available. Indeed, parents envisaged consistent and shared dual career values with athletes, coaches and teachers to determine coordinated efforts in ensuring quality experiences during the holistic development of the student-athletes [23, 36, 40]. Thus, the involvement of parents at academic and sport levels could be enhanced to ensure a coherent supportive entourage for the student-athletes. In considering that in Europe sociocultural expectations of athletes valuing education might differ across countries [29], between educational institutions within a single country [79], between sports organizations [1], and within families, a dual career educational programme for parents could contribute to empower parental support of student-athletes and to foster a dual career culture, especially valued in Member States where the combination of education and sport is still in its developmental phase [2–5, 8].

## Supporting information

S1 File (XLSX)
